# Spectrotemporal content of human auditory working memory represented in functional connectivity patterns

**DOI:** 10.1038/s42003-023-04675-8

**Published:** 2023-03-20

**Authors:** Jyrki Ahveninen, Işıl Uluç, Tommi Raij, Aapo Nummenmaa, Fahimeh Mamashli

**Affiliations:** 1 Athinoula A. Martinos Center for Biomedical Imaging, Massachusetts General Hospital, Charlestown, MA USA; 2 Department of Radiology, Harvard Medical School, Boston, MA USA

**Keywords:** Cognitive neuroscience, Cortex, Human behaviour

## Abstract

Recent research suggests that working memory (WM), the mental sketchpad underlying thinking and communication, is maintained by multiple regions throughout the brain. Whether parts of a stable WM representation could be distributed across these brain regions is, however, an open question. We addressed this question by examining the content-specificity of connectivity-pattern matrices between subparts of cortical regions-of-interest (ROI). These connectivity patterns were calculated from functional MRI obtained during a ripple-sound auditory WM task. Statistical significance was assessed by comparing the decoding results to a null distribution derived from a permutation test considering all comparable two- to four-ROI connectivity patterns. Maintained WM items could be decoded from connectivity patterns across ROIs in frontal, parietal, and superior temporal cortices. All functional connectivity patterns that were specific to maintained sound content extended from early auditory to frontoparietal cortices. Our results demonstrate that WM maintenance is supported by content-specific patterns of functional connectivity across different levels of cortical hierarchy.

## Abstract (web summary)

Functional MRI data obtained during an auditory working memory (WM) task, shows that maintenance of WM in humans involves contentspecific functional connectivity across different cortical hierarchy.

## Introduction

Neuronal processes that help maintain information in working memory (WM), a function critical for our goal-directed behavior, are a long-standing topic of debate. Initially, WM content was thought to be maintained by a dedicated, modular storage circuit. This approach led to a discrepancy in the literature as to whether these modular storage circuits are governed by higher association areas^1–4^ or sensory cortices^5–8^. Those favoring higher areas including prefrontal (PFC) and/or posterior parietal (PPC) cortices argue that representations in sensory areas are too prone to distraction to support stable WM maintenance^9^. The proponents of the “sensory recruitment model of WM”, in turn, note that activations in, e.g., PFCs often correlate more strongly with attentional rather than maintenance-related task demands, per se^8^. Adding to this complexity, a growing body of studies have found evidence for content specific representations from both sensory and association areas during WM maintenance^10–18^.

To address these discrepancies, a synthesis of the competing modular theories is therefore emerging, which suggests that WM maintenance can be distributed to different hierarchical levels whose predominance depends on the complexity of the task and memory items^19–22^. For example, auditory, visual, and tactile memoranda could be represented in parallel in sensory and association areas when the maintained item encompasses both basic sensory and abstract features^16,23^. However, whether and how these parallel distributed representations of maintained WM items interact and are integrated with each other has so far remained an open question^22^.

**Fig. 1:**
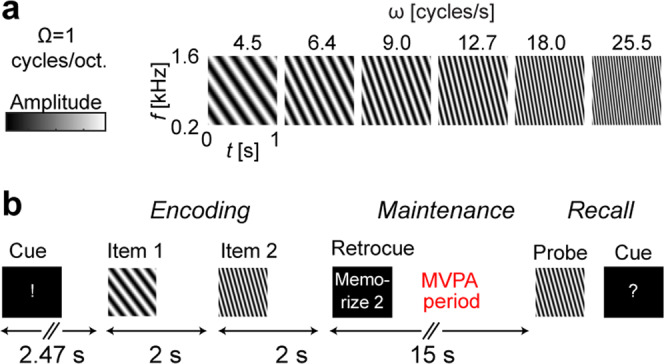
Auditory WM stimuli and task. **a** Time-frequency representations of a prototypical WM set of 6 different moving ripple sounds, modulated across time (ripple velocity, ω cycles/s) and frequency (Ω cycles/octave). **b** Trial design. After a visual preparatory cue, subjects heard two ripple sound stimuli (i.e., potential WM items) in a row. A brief visual retrocue then followed, to instruct which of the previous two items was to be actively memorized for a period of 15 s (“Maintaenance”). After hearing the probe, the subject was asked to press one button (“yes”) if the probe matched the relevant item, and another (“no”) if it did not. The different MVPA analyses were conducted during the Maintenance period (for details, see Methods).

One theoretical possibility is that distributed representations of WM are coordinated via long-range functional connectivity within PFC, PPC, and sensory brain areas^24^. In line with this suggestion, fMRI studies have provided evidence for correlations between behavioral WM performance and the strength of functional connectivity between frontal and posterior brain areas^25^. Whole-brain resting-state fMRI functional connectivity patterns have, in turn, been recently reported to predict individual differences in WM capacity^26^. Accumulating neurophysiological evidence from human^27–29^ and non-human primate studies^30,31^ also suggests that long-range synchronization of neuronal oscillations between brain regions is, in itself, modulated by WM task demands including memory load. While these earlier studies did not probe distributed content representations, a recent human fMRI multivariate pattern analysis (MVPA) demonstrated that the categorical type of WM task demands (e.g., spatial, numeric, or fractal) can be classified based on changes of functional connectivity^32^. Long-range oscillatory synchronization patterns that carry WM information were also found in our recent study that used MEG, a method with a high spectrotemporal resolution but with spatial limitations for mapping of how feature tuning evolves between adjacent cortical areas^33^. Whether stable parametric WM representations could be coded to content-specific connectivity patterns across different levels of cortical feature topography (see, e.g., Fig. 1), thus, remains an open question.

**Fig. 2:**
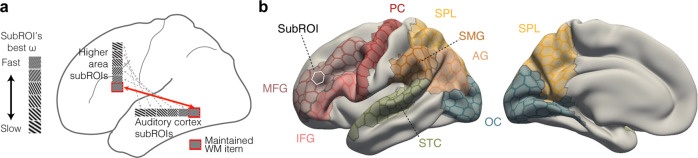
Functional connectivity-based perspective on WM. **a** A hypothesis of how spectrotemporal modulation features such as the “ripple velocity” or *ω* are represented in auditory WM. Different subregions of auditory cortex located in STC, as well subregions of higher frontoparietal areas connected to each subregion of auditory cortex^44–46,61,77^, could show best sensitivity to different *ω* values. The connectivity patterns between STC and frontoparietal areas responsive to ripple sounds could, thus, be arranged according to the prefrred or “best” *ω* (gray dotted arrows)*.* We specifically hypothesized that functional connectivity between STC and frontoparietal areas could be modulated in content specific fashion during WM maintenance (red arrow)^16,33^. **b** Regions of interest (ROI) for our connectivity-based MVPAs testing our main hypothesis. The eight major ROIs are shown on a semi-inflated standard brain surface (left hemisphere, lateral and medial views). Each major ROI was further divided to subROIs (average area 157 mm^2^). The ROI-to-ROI connectivity patterns were defined as the connectivity matrices from across all their subROIs, analogously to our previous MEG studies^33,48,49,91^. Occipital cortex (OC) was included as a control area. The details of ROI definitions and analysis procedures are provided in Methods. Abbreviations not specified above: IFG inferior frontal gyrus, MFG middle frontal gyrus, PC precentral cortex, SPL superior temporal lobule, SMG supramarginal gyrus, AG angular gyrus.

Here, we examined connectivity-based coding of maintained information in the domain of auditory WM, a function much less intensively studied than its visual counterpart, despite its fundamental importance for our everyday communication and behavior^34^. Everyday auditory objects such as vocalizations, pieces of music, and environmental sounds are distributed broadly across time and spectrum, lasting up to several seconds, which could increase the brain’s need to orchestrate its function across hierarchical processing levels^35^. While many auditory studies have so far concentrated on verbal or other cognitively categorizable materials^36–39^, how basic sound attributes are represented in WM has remained relatively little studied. Therefore, here, auditory WM was investigated using parametrically varied dynamic ripple sound stimuli, which are spectrotemporally similar to speech but lack linguistic or categorical labels (Figs. 1, 2). Our hypothesis was that WM content is not only maintained in multiple areas, but that different areas across the processing hierarchy work together in WM retention. To test this hypothesis, we used MVPA to decode the content of auditory WM from fMRI functional connectivity patterns between sub-regions of superior temporal, parietal, and frontal cortices.

## Results

**Fig. 3:**
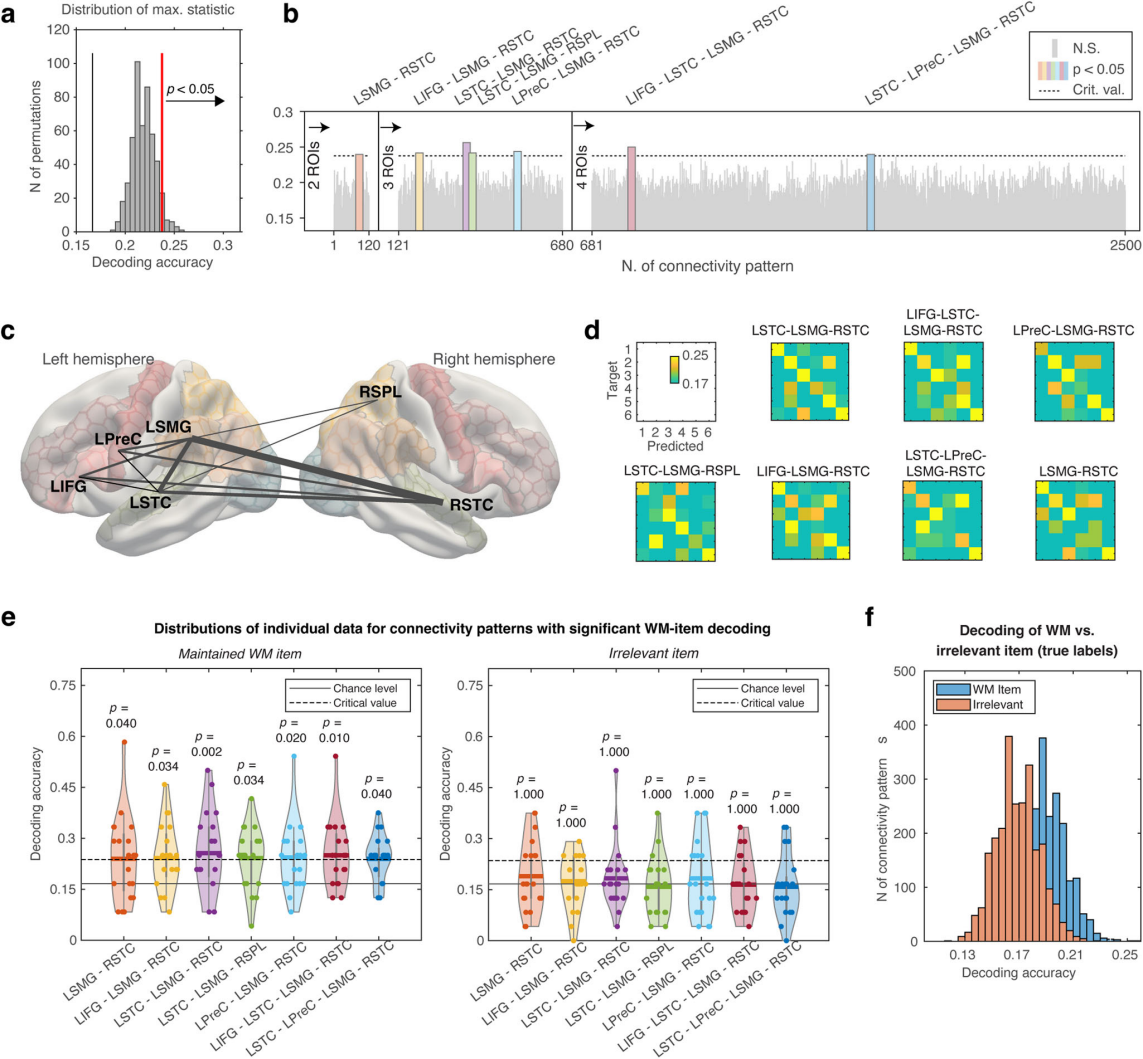
WM content decoded from functional connectivity patterns. **a** The null distribution of maximum statistics across all two- to four-ROI connectivity patterns, generated using classifiers with randomized item labels. **b** Connectivity-based decoding accuracies. WM content was decoded significantly above chance level from 7 out of the 2500 functional connectivity patterns, shown as the colored bars. The narrow gray bars reflect functional connectivity patterns with statistically non-significant decoding accuracies. **c** Anatomical distribution functional connectivity patterns revealing WM content. Notably, left and/or right hemispheric auditory areas of STC were present in all content-specific connectivity patterns. **d** Normalized confusion matrices corresponding to the content-specific functional connectivity patterns, arranged according to their statistical significance level. **e** Distributions of individual data (dots atop the “violin plots”) in the 7 connectivity patterns with significant results for the retro-cued (i.e., actively maintained) “WM item” and non-cued “irrelevant item” (for task details, see Fig. 1). **f** Distributions of group-mean decoding accuracies for the WM item and irrelevant item. Abbreviations: LIFG left inferior frontal gyrus, LPreC left precentral area, LSMG left supramarginal gyrus, LSTC left superior temporal cortex, RSPL right superior parietal lobule, RSTC right superior temporal cortex.

Our MVPA analyses suggest that spectrotemporal attributes of auditory WM can be classified from fMRI functional connectivity patterns between sub-regions of auditory, parietal, and frontal cortices. Auditory cortical areas of superior temporal cortex (STC) were involved in all networks where the decoding accuracy reached a statistical significance according to our non-parametric permutation test (Fig. 3). In addition to the connectivity-based analysis, the content of auditory WM was also decodable from bihemispheric STC and ventral precentral areas using activation-based MVPA.

Auditory WM was examined using a “retro-cueing” paradigm^12,18,33,40–43^, designed to dissociate the differing accounts of recent stimulus history and actively maintained WM content (Fig. 1). Behaviorally, the subjects were able to perform the task according to the instruction, at 83 ± 10% response accuracy (mean ± SEM).

### Evidence for connectivity-based coding of auditory WM

Previous studies have shown that perceptual sensitivity to sound stimuli with differing spectral and temporal modulation pattern varies both across adjacent parts of auditory cortex, as well as across different auditory processing stages and across the two hemispheres^44–46^. We therefore hypothesized that maintaining parametric attributes of auditory stimuli in WM is based on functional connectivity across different parts of this distributed network (Fig. 2a). To test this hypothesis, we trained support vector machine (SVM) classifiers to decode ripple velocity that was maintained in WM during our retro-cueing task from patterns of functional connectivity between a set of regions of interest (ROI). Our ROIs encompassed areas that have been previously associated with auditory and auditory-verbal WM, including STC, posterior parietal, and precentral/lateral frontal areas^12,13,16,47^ (Fig. 2b), as well as occipital cortices that were included as a control area presumed to have no major involvement in auditory WM^33^. Each of these broader ROIs was divided to smaller subunits or “subROIs”^48,49^: The input features to our SVM classifiers consisted of combinations of connectivity matrices across the subROIs between pairs of broader ROIs (Fig. 2b). Using connectivity patterns between sets of subROIs within larger ROIs, as opposed to analyzing all possible subROI-combinations, was utilized to reduce the number of possible combinations and, subsequently, to make it easier to interpret the roles of functional connectivity patterns in maintaining WM representations distributed across the processing hierarchy (see, e.g.^20^).

Using our functional connectivity based MVPA approach, we compared our six-class decoding accuracies to a null distribution calculated by permuting the true labels of the classifier 500 times across all possible 2,500 two, three, and four-ROI functional connectivity patterns (Fig. 3a). According to this analysis, the WM content could be classified significantly above chance level (i.e., 1/6) from seven specific functional connectivity patterns. These functional connectivity patterns were dominated by brain areas known to be involved in perceptual processing of ripple sounds, as well as other auditory spectrotemporal modulation patterns including speech signals (Fig. 3b).

Consistent with theories suggesting that sensory areas play a crucial role in WM, the left, right, or bilateral auditory STC areas as well as the left SMG were involved in all functional connectivity patterns that carried information about the maintained sound content (Fig. 3b). The bilateral STCs and the left SMG also formed the three-area functional connectivity pattern that yielding the highest and statistically most significant decoding accuracy of all studied two- to four-ROI networks (Mean ± standard error of mean, SEM, accuracy 0.26  ±  0.022 ; *p* = 0.002, maximum-statistic permutation test). The numerical details of the other 6 connectivity patterns are detailed in Supplementary Table S1. Other areas occurring in more than one functional connectivity pattern included the left inferior frontal gyrus (IFG) and the left precentral cortex (PreC). Beyond STCs and the left-hemispheric speech processing network, statistically significant decoding accuracies were found also in functional connectivity patterns involving the right superior parietal lobule (SPL).

We also examined the decoding of the ripple velocities of the irrelevant items that were to be forgotten after the presentation of the retro cue. In the connectivity-based decoding analysis, all decoding accuracies remained non-significant according to a non-parametric permutation test that was calculated analogously to the main analysis (Supplementary Results). The results of irrelevant item decoding are compared to those for the retro-cued WM item in Fig. 3e, which shows “violin plots” of distributions across individual subjects in the seven connectivity patterns that yielded significant results in the main analysis. The corresponding numerical values are detailed in Supplementary Table S1. The data in Fig. 3f compares the distributions of group-mean decoding accuracies of the retro-cued VM item vs. irrelevant item, calculated across all studied connectivity patterns with the true (i.e., non-randomized) ripple-velocity class labels. Finally, Supplementary Table S2 shows the results of a control analysis conducted based on the subROI-to-subROI connectivity pattern within each ROI (Fig. 2b). This control analysis yielded no statistically significant results.

### Decoding accuracy in functional connectivity patterns involving occipital visual areas

Although auditory and visual WM systems are known to interact^50^, the direct functional involvement of visual cortex regions of occipital cortex (OC) in auditory WM of ripple sound parameters should be considerably weaker than that of STCs^33^. Functional connectivity patterns including OCs but excluding STCs were therefore used as control networks in our decoding analyses. Among the 2500 functional connectivity patterns in total studied here, one or both OCs were included in 1044 patterns. Of these functional connectivity patterns, 675 were such that they included one of the OCs but neither the left nor right STCs. The decoding results of all these 675 functional connectivity patterns were clearly non-significant, with the best decoding accuracy in functional connectivity patterns involving OCs but no STCs equaling to the median of the null distribution (permutation-based *p* = 0.5).

### BOLD activation based MVPA

**Fig. 4:**
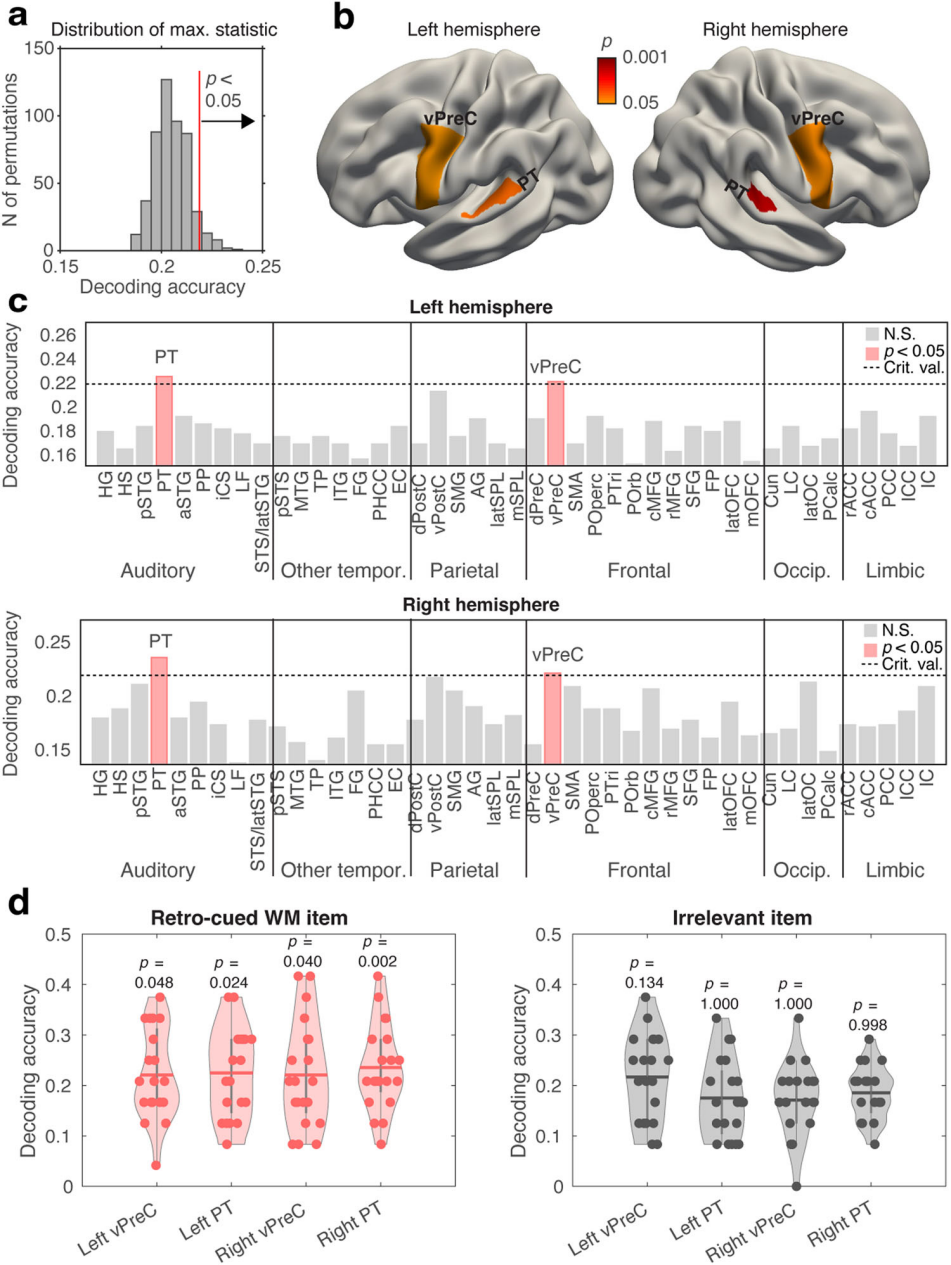
Activation-based MVPA of WM content. **a** The null distribution of maximum statistics for the retro-cued (i.e., actively maintained) WM item across all left and right-hemisphere ROIs, created using classifiers with randomized item labels. **b** ROIs with decoding accuracies significantly above chance level for the retro-cued WM item, mapped onto the “semi-inflated” standard brain representation. WM content could be predicted from bilateral posterior non-primary auditory cortices (left and right PT), as well as from bilateral ventral precentral areas involved in articulatory motor control (left and right vPreC). **c** Decoding accuracies in all 86 ROIs (retro-cued WM item). The ROIs where the decoding accuracy exceeded the critical value determined from the null distribution are shown labeled with pink color. **d** Distributions of individual data (dots atop the “violin plots”) in ROIs with significant results for the retro-cued WM item and non-cued “irrelevant item” (for task details, see Fig. 1; Anatomical abbreviations not defined here are spelled out in Fig. 6).

Our more conventional decoding analysis used an SVM approach, which employed a surface-based ROI decoding with robust non-parametric permutation approach to determine statistical significance. Our surface-based ROIs were determined using a hybrid of FreeSurfer Desikan and Destrieux atlases, modified specifically for comparing the decoding accuracies in auditory-related STC areas to the rest of the cortex. To deal with multiple comparison problems, we compared the activation-based SVM results in each ROI to a null distribution calculated by permuting the true labels of the analysis 500 times across all possible 86 left and right-hemisphere ROIs included in the analysis (Fig. 4a). At the group level, the decoding accuracies were statistically significantly above chance in bilateral posterior non-primary auditory cortices of STC and ventral precentral (PreC) regions that overlap the motor and pre-motor regions controlling articulatory-motor functions. The four ROIs with significant results according to our non-parametric permutation test included the left planum temporale (PT), the right PT, as well as the left PreC and the right PreC (Fig. 4b, c). No statistically significant results were found in an analogous SVM analysis for the task-irrelevant item.

Control analyses of fMRI data using surface-based univariate GLM are described in Supplementary Material **(**Supplementary Results). Examples of the results are shown in Supplementary Fig. S1.

## Discussion

Using machine-learning techniques, we demonstrate that parametric attributes of auditory working memory can be classified from distributed patterns of fMRI functional connectivity. To determine statistical significance of decoding accuracies at the group level, we used non-parametric permutation testing where the results were compared to a null distribution that considered all possible two, three, or four-ROI connectivity patterns within a bilateral frontoparietal-temporal network. This robust analysis suggested that different levels of ripple velocity, a fundamental parameter of speech and other natural sounds, are represented in WM by combinations of functional connections across auditory, premotor, and frontoparietal association areas. Despite the non-verbal nature of our stimuli, these content-specific connectivity patterns were concentrated in the left hemisphere, overlapping with pathways believed to govern maintenance through subvocal rehearsal within the “phonological loop” of human WM^51^. Networks involving occipital cortex, a control region presumed to lack any fundamental roles in auditory functions, did not reveal significant decoding results. The decoding results for the irrelevant item, which was to be forgotten after the retro-cue, were statistically non-significant, supporting the interpretation that the connectivity-based MVPA results reflect WM instead of a passive sensory buffer. It has been long argued that WM information can be distributed to different levels of the processing hierarchy^20^. Based on the present findings, we propose that memorized content is not only represented at multiple hierarchical levels, but that different areas across the processing hierarchy work together to maintain WM.

The present findings could have direct relevance for the broader theoretical debate of how and where in the human brain information is maintained during WM processing, beyond the auditory domain alone. The classic theory, which is founded on the idea that WM information is maintained in a dedicated storage circuit (for recent arguments, see^52,53^), is being increasingly challenged by evidence that WM content is decodable from multiple brain regions, ranging from early sensory to highest association areas^10–17,20,47,50,54,55^. Distributed models of WM consequently suggest that instead of a dedicated single region, different attributes of the same WM item could be represented in parallel in multiple areas, depending on their complexity, the degree of abstraction, and the level of precision required to support their maintenance^20,22^. Whether and how the different hierarchical levels of the same WM representations interact and are integrated has, however, remained uncertain^22^. The evidence supporting distributed models has concentrated on studies, which have shown that information of a given cognitive attribute may be decodable from multiple, anatomically separated brain areas. This has left room for an interpretation that MVPA findings at lower levels, including sensory cortices, reflect epiphenomenal feedback from a higher maintenance module in PPC or PFC^9^. It is thus important to note that in the present study, all functional connectivity patterns with significant decoding accuracies of WM content spanned multiple hierarchical levels between sensory and association areas. In the light of this finding, stable representations of maintained content can be distributed to multiple brain regions at different processing levels to support maintenance of WM. Our working hypothesis for future studies is that WM maintenance is an emergent property of such a connectivity-based coding scheme: No single area is necessarily in the sole control of maintenance^56^.

Hierarchically distributed maintenance of ripple sounds, which are dynamic multifeature patterns unfolding over the course of hundreds of milliseconds, is consistent with evidence that neurons sensitive to sensory attributes of complex sounds occur at multiple processing levels^35,57,58^. For example, the non-human primate (NHP) homolog of human IFG includes neurons that support perceptual discrimination of purely acoustic morphological patterns of auditory stimuli^57,59^. Distributed networks spanning multiple processing levels could be elementary not only to WM, but also to our ability to perceive the temporal structure of auditory objects that may consist of multiple events that progress over time^35^. Consistent with these notions, previous studies show that neuronal populations sensitive to different spectrotemporal modulation rates exist at multiple levels of auditory processing hierarchy^44,45,60,61^. At the same time, the preferred amplitude and/or frequency modulation rates may vary between subareas of auditory cortex, between the different hierarchical levels, and also between pathways in each brain hemisphere^44,45,60,61^. The content-specificity of functional connectivity patterns during WM maintenance could, thus, reflect an intrinsic connectivity topography across subareas, which are located at different processing levels but maintain representations of similar ripple velocities (Fig. 2a). Indices of such an arrangement were found in our earlier study using MEG^33^, a method that offers high spectral/temporal resolution but is less optimal for detailed mapping of cortical feature topography than our current fMRI approach.

In the visual domain, a key argument against distributed models, which posit a role also for sensory cortices, has been that areas that are activated strongly by the stimuli themselves are too prone to distraction to support WM^52^. In the present study, such low-level distraction was provided by the acoustical scanner noise, which contains modulations somewhat similar to the to-be-maintained ripple stimuli. Yet, auditory-cortical areas of STC were included in all functional connectivity patterns informative of WM content. Further, in the activation-based MVPA, the content of auditory WM could be also decoded significantly above the chance level from the bilateral posterior non-primary auditory cortex areas, consistent with earlier studies using amplitude modulated sounds^16^.

In our connectivity-based MVPA, the information-containing patterns involved functional connectivity across heterotopic interhemispheric areas, the most prevalent being the connection across RSTC and LSMG. This might be suprising given that inter-hemispheric transfer of information is, generally, dominated by homotopic anatomical connectivity, being evident also in fMRI functional connectivity studies^62^, including the present control analyses (Suppl. Fig. S1). One possible explanation for the present result is that the significant MVPA results involving heterotopic connectivity patters reflect multi-synaptic connectivity that is mediated via a third area (e.g., LSTC in the case of RSTC-LSMG pattern) whose role was not detected due to limitations such as noise or lack of sensitivity. However, recent human post-mortem^63^ and multi-species mapping studies^64^ suggest that heterotopic connectivity may play a more significant role in cognition than previoiusly thought. For example, post-mortem studies in humans suggest that LIFG, one of the areas involved in information-containing heterotopic connectivity patterns with RSTC in the present study, has direct monosynaptic connections with sensory areas of the right hemisphere^65^. Furthermore, fiber tracing studies in NHPs suggest heterotopic connections that extend from non-primary auditory areas of STC to the opposite frontal and parietal cortices^66^. For example, LSMG is thought to be a nodal point of auditory WM networks^67–69^: spectrotemporal information processed in the RSTC might need to be linked to this hub via heterotopic connectivity to support auditory WM. Further studies on the role of heterotopic inter-hemispheric pathways in coding of complex spectrotemporal patterns in human auditory WM are, thus, clearly warranted.

The activation-based MVPA showed significant WM decoding results in bilateral posterior non-primary auditory areas, as well as in the left ventral precentral cortex. These results are generally consistent with results obtained with a slightly different modulation patterns using a three-dimensional fMRI searchlight decoding analysis^16^, as well as with MVPA studies using pure-tone material^12^. The sligth differences between the present and previous results, such as the lack of significant results in the left IFG^12^, could reflect methodological differences between the studies (e.g., different ROIs, search strategies strategies, and statistical approaches). It is worth noting that many of the areas involved in information containing ROI-to-ROI patterns of the connectivity-based MVPA did not yield significant results in the activation-based analysis. One potential explanation is that not only auditory-cortical, but also precentral ROIs that are heavily connected with STC to support auditory perception and production of speech^70^, could contain larger proportions of neurons receiving direct sensory input at the encoding stage. This could have increased the sensitivity of activation-based MVPA in auditory cortices and precentral ROIs than the other ROIs.

Another potential limitation of the present study is that the screening of participants was based on self-reported history of hearing difficulties and risk factors such as exposure to loud noises at work, instead of audiometic assessment of pure-tone thresholds. However, this potential limitation is mitigated by the fact that the stimulus materials were adapted to each volunteer’s ripple-velocity discrimination thresholds. As for the theoretical generalizability of our connectivity-based results, another limitation is that the stimulus material consisted of auditory stimuli alone. Given the temporally distributed nature of auditory objects, the role of long-range connectivity in maintaining of comparable visual stimuli, such as Gabor patches, might not follow similar principles. An inherent limitation of using fMRI to examine functional connectivity is that the lack of temporal resolution provides limited means for examining the exact temporal order of events or causal roles of different areas within the multi-regional connectivity patterns containing auditory WM information in the present study.

In conclusion, our results demonstrate that sensory information maintained in auditory WM can be decoded from fMRI functional connectivity patterns between subregions of early sensory, posterior parietal, and frontal cortices. This result suggests that auditory WM information is not only decodable from multiple hierarchical levels, but that brain areas across the processing hierarchy work in concert to support WM representations.

## Methods

### Subjects

The study was based on data from 20 healthy right-handed participants (12 females, ages 22–47 years) with self-reported normal hearing. The data of two participants of an initial sample of 23 were excluded due to the difficulty in performing the task (proportion correct 0.41 and 0.54), and one subject’s data were excluded due to a triggering problem between the scanner and stimulus presentation computer. The protocol of the imaging experiment was approved by the Partners Human Research Committee, the Institutional Review Board (IRB) of the MGH. All participants gave a written informed consent before participating in the study.

### Stimuli and task

Many previous studies on auditory WM have used stimuli that allow non-auditory maintenance strategies^36–39^. Here, we utilized moving ripple sounds, which are broadband sound patterns modulated across time (ripple velocity, ω cycles/s) and frequency (Ω cycles/octave) (Fig. 1). Moving ripple sounds are spectrotemporally similar to speech^71^ but not contaminated by semantic properties or perceptual categories^72^. This helps eliminate verbal and other non-auditory rehearsal strategies. The WM items consisted of 1-s sounds with six ripple velocities separated by 1.5 of each individual participant’s just noticeable difference (JND). The JND was determined for each participant in a separate session to control for differences in sound discrimination^73^. To obtain the stimuli, for each participant, we first created an individualized set of 17 stimuli with different ripple velocities, separated by intervals of **Δ**ω = 0.5 × JND. JND was approximated as the minimally detectable base 2 logarithmic ripple-velocity interval within a range of 3–48 cycles/s based on an adaptive 1 down/ 2 up staircase algorithm. The moving ripple sounds were generated by superimposing 20 random-phase sinusoids/octave ranging from *f*_0_ = 0.2 kHz to *f* = 1.6 kHz. Their intensity at any time and frequency is defined by *s(g,t*) *=* *D*_*0*_ + *D·*cos[2π**(***ωt*  +  *Ωg*) *+* *ψ*], where g is log(*f/f*_*0*_), D is the modulation depth, and *ψ* is the phase of the ripple (sound duration = 1 s, Ω = 1 cycles/octave, the lowest possible ω = 4 cycles/s).

The sound stimuli were delivered via an MRI compatible Sensimetrics S14 system (Sensimetrics, Gloucester, MA) and the visual stimuli via a video projector and mirror system. The stimuli were presented and behavioral responses collected using a Dell Precision 3000 M3510 laptop computer (Dell Technologies, Round Rock, TX), which was equipped with an external Soundblaster XFI HD soundcard (Creative Technology Ltd., Jurong East, Singapore). The paradigm was run by Presentation software (Neurobehavioral Systems, Berkeley, CA) synchronized with the fMRI volume acquisitions via its USB port.

Auditory WM was examined using a “retro-cueing” paradigm^33^, modified from recent auditory^12,16,18,40^ and visual^41–43^ WM studies (Fig. 1). The benefit of this design is that it helps control for the differing accounts of recent stimulus history and actively maintained WM content. In this design, the subject was first presented with two sound items in a row. A subsequent “retrocue” will indicate which of the two items is to be maintained in memory. The subject will press one button if the probe matches the relevant item and another if not. The simple matching task was selected to minimize the usage of non-auditory strategies. In 50% of the trials the probe matched the relevant item. Half of the remaining trials (25% of the total count) were non-match trials where neither of the two items matched the target, and in the rest of the trials (25% of the total count) the irrelevant item matched the probe. The potential memory items consisted of only 6 possible classes, whereas the probes were selected from the entire individualized pool of 17 possible stimuli. Participants were naïve to the number of memory items presented to them to prevent categorization. To increase the physical variability, there was a half-JND offset between the possible relevant vs. irrelevant items. In total, the task consisted of four runs, each with 24 trials (4 trials per each item class).

### Data acquisitions

High-resolution T1-weighted anatomical images were obtained using a multi-echo MPRAGE pulse sequence (TR = 2530 ms; 4 echoes with TEs = 1.69, 3.55, 5.41, 7.27 ms; 176 sagittal slices with 1 × 1 × 1 mm^3^ voxels, 256 × 256 mm^2^ matrix; flip angle = 7°)^74^ in a 3 T Siemens Prisma whole-body MRI scanner (Siemens Medical Systems, Erlangen, Germany) using a 64-channel head and neck coil. fMRI data were obtained with a gradient-echo (GE) EPI sequence, TR/TE = 1,470/30 ms, flip angle = 82°, iPAT 2, SMS 3, 2 × 2 × 2 mm^3^ voxels; 69 axial slices.

### Basic data analyses

Behavioral performance was determined as the proportion of correct responses.

#### MRI and fMRI preprocessing

Cortical surface reconstructions, anatomical normalizations, and fMRI analyses were conducted using Freesurfer 6.0^75,76^. For the MVPA analyses, fMRI volumes were motion corrected to a session-based template, realigned temporally to correct for slice timing differences, coregistered with structural MRIs, and intensity normalized. Distortions from *B*_0_ field inhomogeneities were compensated by EPI unwarping. After preprocessing, the data were entered into a general-linear model (GLM) with the task conditions as explanatory variables. In all analyses, the design matrix also included physiological and motion regressors of no interest, as well as polynomial regressors corresponding to a high-pass filter with a cutoff frequency of 0.006 Hz to remove low-frequency drifts in the BOLD signal. Further details are specified in the context of our different decoding analyses below.

### Connectivity-based MVPA

#### Theoretical rationale

Inspired by distributed models of WM maintenance, we hypothesized that ripple-sound content is maintained broadly across areas sensitive to auditory spectrotemporal modulation patterns, as demonstrated in previous studies on auditory perception^44–46^ or auditory WM processing^16,33^ (Fig. 2a). The sensitivity to particular parameters of spectrotemporal modulation patterns differs between subregions of auditory cortex^44,46^, between hemispheres^61^, and along the posterior-anterior object processing hierarchy that extends from STC to higher areas including IFG^77^. Furthermore, medial aspects of STC that are closer to primary auditory cortex are sensitive to higher temporal modulation rates than lateral STC^46^. The left hemisphere could be sensitive to finer temporal modulation rates than the right, within and beyond auditory areas of STC^45,61^. Based on this diversity of spectrotemporal tuning properties, we hypothesized that WM processing of different ripple velocities could recruit a distributed network extending from bilateral STCs to parietal and frontal areas.

#### Regions of interest (ROI)

To test our connectivity-based hypothesis, we defined a set of broader ROIs reported previously to play a role in auditory, verbal, or other aspects of WM^1,16,33,39,51,78–81^ (Fig. 2b). The idea was that content-specific coding of WM information would be revealed based on the pattern of functional connectivity across the different subareas of these larger ROIs. Each of these broader ROIs was thus divided to multiple smaller subROIs whose average surface area across all subjects and areas was 157 mm^2^. It was our assumption that pooling together the signals to slightly larger subROIs would not only increase the computational efficiency and reduce the number of features in the decoding analysis, but also increase the SNR of the features.

Each subROI of the larger ROIs referred to the icosahedral patches corresponding to the vertices of the fsaverage3 standard brain (642 vertices / hemisphere), resampled to each individual subject’s higher-resolution cortical representation. The ROIs included superior temporal cortex (STC; superior temporal gyrus and Heschl’s gyrus (HG) combined; *n*_subRois_ = 34 left/29 right), middle frontal gyrus (MFG, rostral, caudal parts combined; *n*_subRois_ = 43 left/50 right), inferior frontal gyrus (IFG; *n*_subRois_ = 23 left/14 right), precentral cortex (PreC), supramarginal gyrus (SMG; *n*_subRois_ = 34 left/32 right), angular gyrus (AG; *n*_subRois_ = 33 left/37 right), and superior parietal lobule (SPL; *n*_subRois_ = 76 left/80 right) (Fig. 2b). STC, SMG, AG, PreC, and IFG were chosen as ROIs because of their presumed (left-hemisphere dominant) role as the anatomical substrate of the “phonological loop”^51,78,82^, which was proposed to support maintenance of auditory-verbal items in the classic model^83^. On the other hand, areas overlapping with *bilateral* STC, SMG/AG, and PC have also been implicated in WM maintenance of non-verbal attributes such as auditory amplitude modulation (AM) rates^16^. An earlier MVPA study, in turn, suggested that distributed activation patterns of IFG and STC support WM maintenance of sound frequency^12^. MFG, which is often referred to as dorsolateral prefrontal cortex (DLPFC), is where WM-specific “maintenance units” were first reported^1^. MFG/DLPFC has been associated with a multitude of different roles in human WM^39,79–81^ and in its clinical dysfunctions^84^. SPL has, in turn, been associated with maintenance of auditory-spatial location content in human WM^18^, and it has been also implicated in activity-silent maintenance of visual WM^41^ as well as in cognitive control of WM maintenance^85^. In addition to these frontoparietal and temporal ROIs, we also included the occipital cortex (OC; *n*_subRois_ = 62 left/51 right) to serve as a control area, with the assumption that this area would not play a major role in connectivity-based WM maintenance of *auditory* information^33^.

#### Functional connectivity

**Fig. 5:**
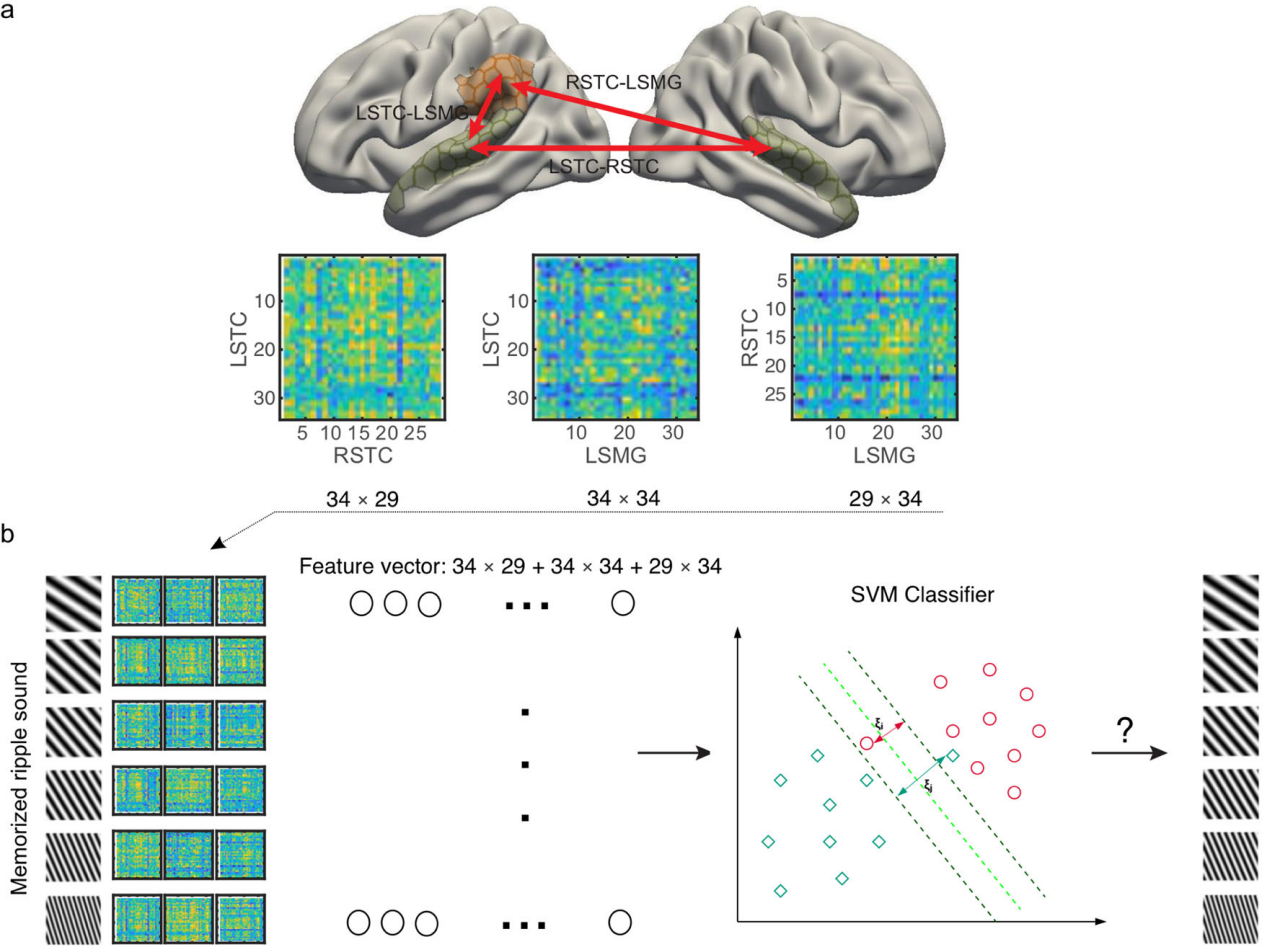
Schematic illustration of the connectivity-based MVPA approach. **a** An example of a functional connectivity pattern across subROIs of left STC, left supramarginal gyrus (LSMG), and right STC. In our analyses, we used all possible two, three, and four ROI functional connectivity patterns across our broader cortical ROIs. Using connectivity patterns between sets subrois within larger ROIs (as opposed to analyzing all possible subROI-combinations) was intended to help conceptualize the potential role of representations spanning different hierarchical stages of WM processing (see, e.g. ref. ^20^). **b** Functional connectivity based decoding approach. The functional connectivity pattern matrices were converted to a vector consisting of 34 × 29 + 34 × 34 + 29 × 34 features, to classify the ripple velocities held in auditory WM.

fMRI functional connectivity patterns between the ROIs were based on the residuals of the task-related GLM calculated in each subjects native functional space, from which the nuisance effects and inter-regional co-activations related to task performance had been regressed out. The following stimulus events were modeled as separate regressors in this GLM: the visual alerting stimulus (“!”), the two successive memory items, and the memorization cue (“Memorize 1” or “Memorize 2”), the probe stimulus, and the visual responding cue (“?”). In addition to motion-related regressors, we regressed out the contribution of signals originating in the cerebrospinal fluid (CSF) and white matter from these residuals. No spatial smoothing was applied. To obtain the subROI-specific residualized time series for the fMRI functional connectivity analysis, we resampled each subject’s unsmootheed time series to the Freesurfer “fsaverage3” standard-brain representation using nearest-neighbor interpolation. For each run, class-specific Pearson correlation matrices were then calculated based on the residualized fMRI time series during the maintenance periods, lagged by 4.41 s and concatenated within classes in each run, across the subROIs of all possible pairs of left and/or right hemisphere ROIs. The resulting connectivity matrices between any two ROIs A and B consisted of *N*_*A*_ × *N*_*B*_ sub-ROI pair connections, giving *N*_*A*_ × *N*_*B*_ = *T*_*AB*_ features, which were reshaped to a vector for the MVPA analyses (Fig. 5).

Our research was guided by the assumption that functional connectivity patterns specific to memorized sound attributes reflect modulations of networks maintaining information in largely activity-silent or hidden processing states. To resolve this challenge, we pursued an idea that the sensitivity of decoding would be improved if we considered patterns of functional connectivity across not only pairs, but also across slightly more complex inter-regional assemblies. In addition to pairs of ROIs, we therefore considered ROI networks that included three or four nodes. In these cases, the connectivity matrices representing all possible pairs of ROIs within the network were reshaped and concatenated to one “functional connectivity pattern” vector. In the case of a three-node functional connectivity pattern across areas A, B, and C, the number of features in this vector was thus $T={T}_{AB}+\,{T}_{AC}+{T}_{BC}$. In the case of a four-node functional connectivity pattern across areas A, B, C, and D, the number of features was $T={T}_{AB}+\,{T}_{AC}+{T}_{AD}+{T}_{BC}+{T}_{BD}+{T}_{CD}$.

#### Machine learning

MVPA analyses were conducted using support vector machine (SVM) implemented in libsvm^86^ and provided in the COSMOMVPA package (http://www.cosmomvpa.org/)^87^ in MATLAB. A SVM classifier with linear kernel and cost equal to one (*C* = 1) was trained using 18 × T dataset (3 of the 4 runs) and tested on a separate 6 × T dataset (the remaining run), employing a leave-one-out four-fold cross validation.

#### Statistics and reproducibility

To control for multiple comparisons, statistical significances of decoding accuracies were tested at the group level using a nonparametric randomization approach. First, we created 500 random permutations where the true labels of the classifier were shuffled within each exchangeability block, i.e., the fMRI runs. To determine the classification accuracies that emerge by chance with 6-classes, a distribution of decoding accuracies using training data with randomized item-content labels was generated across all subjects and connectivity patterns. For the final null distribution, we selected the maximum group mean across all possible connectivity patterns from each permutation. To assign a p-value for each connection, the original group mean accuracy value, found from classifiers with true labels, was compared with this null distribution.

### Activation-based MVPA

#### ROI definition

**Fig. 6:**
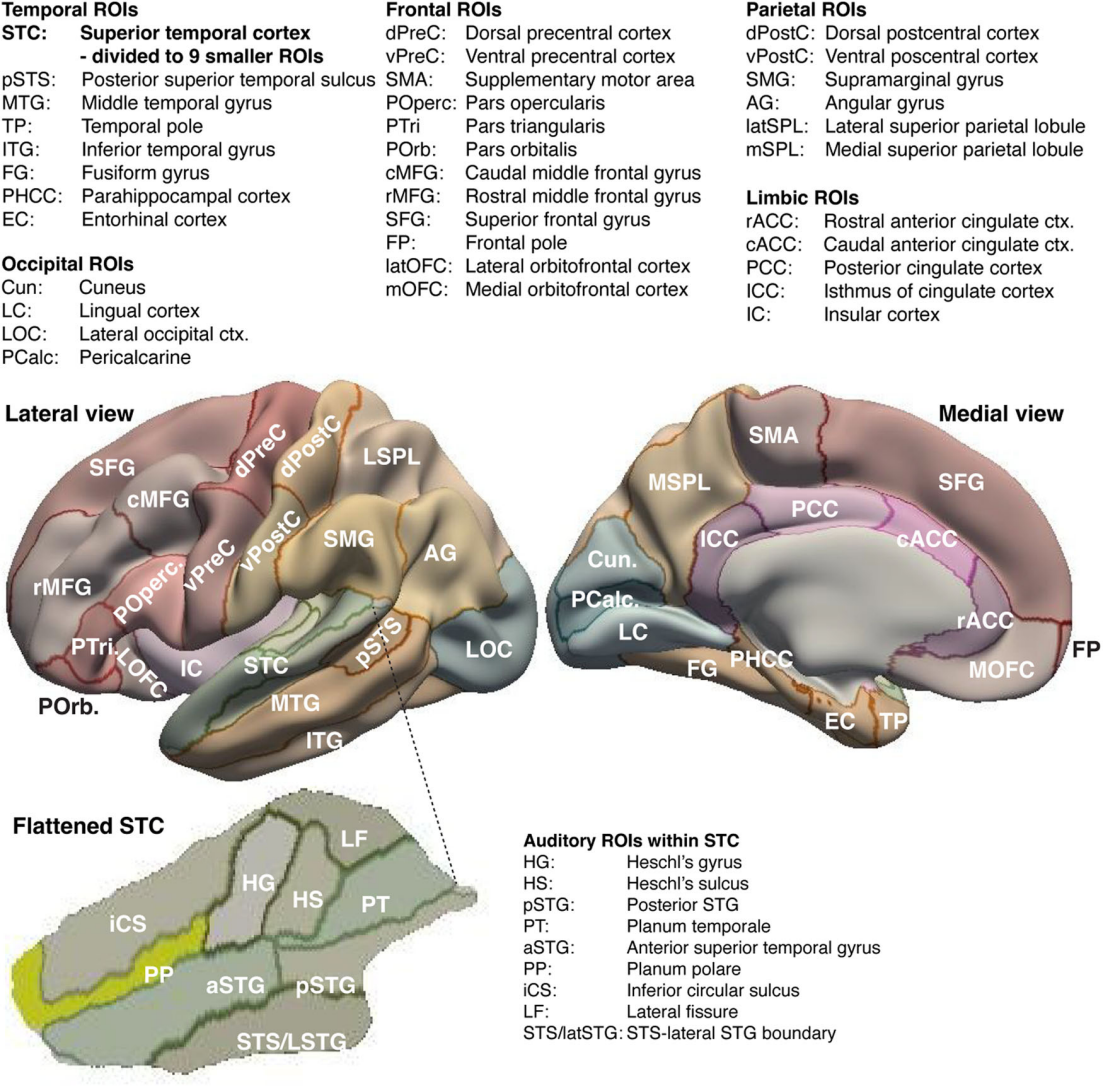
Regions of interest (ROI) in activation-based MVPA analyses. ROIs were defined based a modification of the Freesurfer Desikan atlas, with the auditory-related superior temporal cortex (STC) areas divided to 9 smaller areas based on the more detailed Freesurfer Destrieux atlas (Bottom panel). In each subject, the surface-based atlases were resampled to the unsmoothed native functional space.

The conventional MVPA analyses were conducted in the native functional space with no spatial smoothing: To focus the analyses to the cortical gray matter, and to minimize cross talk across sulci and gyri, a set of a priori anatomical ROIs were defined based on modified Freesurfer surface-based anatomical segmentations, calculated individually using the “recon-all” function of Freesurfer (Fig. 6). A total of 43 surface-based ROI labels per hemisphere were projected to each subject’s unsmoothed native functional space. To define these areas, the Desikan anatomical parcellation^88^ was modified such that the combination of labels encompassing the superior temporal cortex (areas STG and HG) were divided to nine smaller parcels based on the more detailed Freesurfer Destrieux atlas^89^, with the STG further divided to its anterior and posterior portions using mris_divide_parcellation. The STC areas of interest included Heschl’s gyrus (HG), Heschl’s sulcus (HS), planum temporal (PT), posterior STG (pSTG), anterior STG (aSTG), as well as the parts of Destrieux’s lateral fissure (LF), inferior circular sulcus (iCS), and superior temporal sulcus (STS/STG) that overlap with Desikan’s STC. The more detailed parcellation of STC was utilized to pinpoint areas with sharpest auditory-parametric WM representations. In addition, we used mris_divide_parcellation to define the dorsal and ventral subareas of precentral (vPreC, dPreC) and postcentral areas (vPostC, dPostC) to search parametric WM representations specifically in the ventral sensorimotor areas that are presumed to be involved in the “phonological loop” of auditory WM^51^. As determined from the group average sizes for each ROI, the median number of 2-mm isotropic voxels within these ROIs was 805, with the smallest and largest ROIs being the right Heschl’s Sulcus (HS, 86 voxels) and the left superior frontal gyrus (SFG, 4486 voxels), respectively.

#### Preparatory fMRI analysis

After preprocessing (see above), task-related GLM was calculated in each subjects native functional space, with no spatial smoothing. The following stimulus events were modeled as separate regressors: the visual alerting stimulus (“!”), the two successive memory items, and the memorization cue (“Memorize 1” or “Memorize 2”), the probe stimulus, and the visual responding cue (“?”). In addition to these external stimuli, a set of content-specific regressors modeled the effect of ripple-velocity content held in WM during the maintenance period. The content-specific regressors, corresponding to each of the memorized ripple velocities, encompassed the maintenance period starting 4 s after the “memorize” cue onset until the onset of the probe. The GLM was calculated separately for each of the four runs and the resulting contrast effect size estimates (i.e., beta values multiplied by the contrast matrix) were used as features in the MVPA.

#### Machine learning

Activation-based MVPA analyses were conducted using a similar linear SVM classifier to the connectivity-based MVPA, with libsvm^86^ provided in the COSMOMVPA package (http://www.cosmomvpa.org/)^87^ in MATLAB. An SVM model with linear kernel (*C* = 1) was trained using 18 × T dataset (three runs) and tested on 6 × T dataset (1 run), where T refers to the number of voxels in each ROI, employing four-fold cross validation.

#### Statistics and reproducibility

To control for multiple comparisons, statistical significance of decoding accuracies was tested at the group level using a nonparametric randomization approach. First, we created 500 random permutations where the true labels of the classifier were shuffled within each run. To determine the classification accuracies that emerge by chance with 6-classes, a distribution of decoding accuracies using training data with randomized item-content labels was generated across all subjects and ROIs using the same maximum-statistic permutation test procedure as for Connectivity-Based MVPA: For the final null distribution, we selected the maximum group mean across all ROIs from each permutation. To assign a p-value for each connection, the original group-mean accuracy value, found from classifiers with true labels, was compared with this null distribution.

Univariate GLM analyses are described in the Supplementary Information (Supplementary Methods).

### Reporting summary

Further information on research design is available in the Nature Portfolio Reporting Summary linked to this article.

## Supplementary information

Supplementary Information

Reporting Summary

## Data Availability

The data for reproducing the connectivity-based main findings of the paper are available on Harvard Dataverse (10.7910/DVN/51WR3A)^90^. All other data of this study are available from the corresponding author upon reasonable request.

## Code availability

The custom code for reproducing the connectivity-based main findings of the paper are available on Harvard Dataverse (10.7910/DVN/51WR3A)^90^.
