# Supplementary material for: Spectrotemporal content of human auditory working memory represented in functional connectivity patterns
Source: Commun Biol. 2023 Mar 20;6:294. doi: 10.1038/s42003-023-04675-8 (PMC10027691; doi:10.1038/s42003-023-04675-8)
Supplement: Supplementary file 1 — Supplementary Information [file 42003_2023_4675_MOESM1_ESM.pdf]

Supplementary Information

Supplementary Results

Connectivity-based MVPA of irrelevant items. All connectivity-based MVPA results for the irrelevant item, which was to be forgotten upon seeing the retro cue in each trial (for details, see **Fig. 1** and Methods), were statistically non-significant in the maximum-statistic permutation test. **Supplementary Table S1** shows a comparison between the MVPA results for the irrevant item and the retro-cued WM item, within the seven networks yielding significant results for the latter (see also **Figs. 3e**, **3f** of the main text).

For the irrelevant item, the largest (but statistically non-significant) decoding accuracy of 0.227, corresponding to *p*=0.246, was observed for a connectivity pattern involving LMFG, LSPL, RSTC, and the right angular gyrus (RAG), which was not among the patterns yielding significant results for the retro-cued WM item either.

Supplementary Table S1. Group means and standard errors of mean (SEM) of decoding accuracies and p-values based on the maximum-statistic permutation test in the 7 connectivity patterns with significant results (**Fig. 3**). The decoding accuracies of the WM item are compared to those obtained for the uncued, i.e., irrelevant item, which was to be forgotten after the presentation of the retro cue. Abbreviations: LIFG, left inferior frontal gyrus; LPreC, left precentral area; LSMG, left supramarginal gyrus; LSPL, left superior parietal lobule; LSTC, left superior temporal cortex; RSPL, right superior parietal lobule; RSTC, right superior temporal cortex.

	**WM Item**			**Irrelevant Item**		
Connectivity pattern	*Mean*	*SEM*	*p*	*Mean*	*SEM*	*p*
LSMG - RSTC	0.24	0.028	0.04	0.19	0.023	1
LIFG - LSMG - RSTC	0.24	0.022	0.034	0.17	0.018	1
LSTC - LSMG - RSTC	0.26	0.026	0.002	0.18	0.022	1
LSTC - LSMG - RSPL	0.24	0.02	0.034	0.16	0.019	1
LPreC - LSMG - RSTC	0.24	0.022	0.02	0.18	0.025	1
LIFG - LSTC - LSMG - RSTC	0.25	0.022	0.01	0.16	0.019	1
LSTC - LPreC - LSMG - RSTC	0.24	0.016	0.04	0.16	0.021	1

Connectivity-based MVPA within ROIs. The results of an MVPA based on the connectivity-patterns across the sub-ROIs across each ROI are shown in **Supplementary Table S2**. No significant decoding accuracies were found in this analysis.

Supplementary Table S2. Group means and SEMs of decoding accuracies based on within-ROI subROI-to-subROI connectivity for the maintained WM item. Abbreviations: STC, superior temporal cortex; SMG, supramarginal gyrus; AG, angular gyrus; SPL, superior parietal lobule; PreC, precentral area; IFG, left inferior frontal gyrus; MFG, middle frontal gyrus; OC, occipital cortex.

	**Left Hemisphere**			**Right Hemisphere**		
ROI	*Mean*	*SEM*	*p*	*Mean*	*SEM*	*p*
STC	0.17	0.020	1.00	0.17	0.000	1.00
SMG	0.17	0.017	1.00	0.17	0.011	1.00
AG	0.20	0.019	0.43	0.17	0.016	1.00
SPL	0.21	0.014	0.10	0.19	0.022	0.65
PreC	0.19	0.020	0.79	0.18	0.018	0.85
IFG	0.17	0.017	0.99	0.16	0.019	1.00
MFG	0.19	0.019	0.79	0.17	0.017	0.99
OC	0.15	0.018	1.00	0.18	0.017	0.93

Functional connectivity strength. The results of a control analysis of functional connectivity across the ROIs are shown in **Supplementary Fig. S1**. The ROI-to-ROI connectivity matrices were calculated using partial correlations across all possible pairs during WM maintenance (**Fig. S1a**) and during the inter-trial period (**Fig. S1b**, the last two volume acquisitions before the "ready" cue). The analysis was based on the same residualized fMRI time courses that were utilized for the connectivity-based decoding, averaged within each ROI. To display the results, group-averages of Fisher-transformed correlation coefficients were masked to elements with statistically significant results. During maintenance, a larger number of significant frontoparietal connections were observed with bilateral STCs than with OCs (**Fig. 3**). At the same time, the number of significant connections between STC areas and frontoparietal regions appeared to be larger during maintenance than during the pre-cue period. Finally, in both analyses, strong connectivity values were observed between homotopic inter-hemispheric pairs of ROIs (i.e., RSTC-LSTC, LOC-ROC).

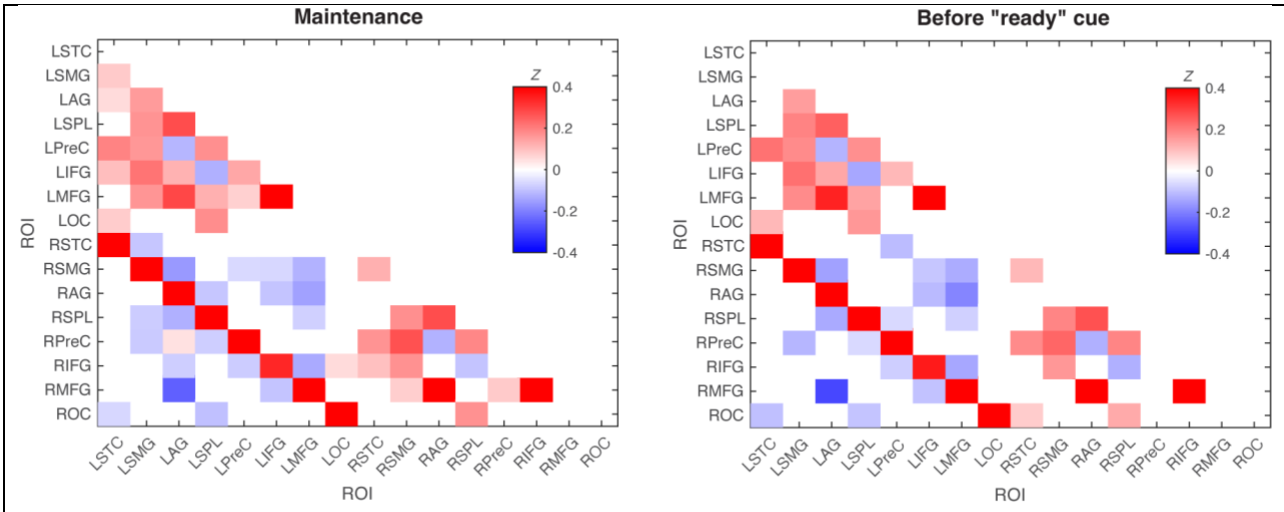

Suppl. Fig. S1. Functional connectivity strengths between ROIs. (**a**) Group-average Fisher-transformed partial correlation coefficients between ROIs during the maintenance period, masked to elements with significant results. (**b**) Group-average Fisher-transformed partial correlations during the pre-trial period, masked to elements with significant results. Partial correlations were calculated between the ROI-averages of the residualized fMRI time courses that were also used for the connectivity-based MVPA areas. The statistical significance was determined using element-wise paired t-tests corrected for multiple comparisons using the false discovery rate (FDR) procedure of Benjamini and Hochberg (Benjamini and Hochberg, 1995).

Activation based MVPA for irrelevant items. No statistically significant decoding results were obtained in the activation-based MVPA for the irrelevant items, which were to be forgotten after the presentation of the retrocue (for details, see **Fig. 1** of the main text). The ROI with the largest decoding accuracy was found in the Left vPreC (Mean = 0.217, SEM = 0.019, p=0.134). **Supplementary Table S3** compares decoding accuracies in the activation-based MVPA for the retro-cued (actively maintained) WM item and the irrelevant item in the four ROIs where the results for the WM item were statistically significant.

Supplementary Table S3. Activation-based MVPA results for the WM item and irrelevant item in the four ROIs with significant results for the former.

	**WM Item**			**Irrelevant Item**		
ROI	*Mean*	*SEM*	*p*	*Mean*	*SEM*	*p*
Left vPreC	0.221	0.02	0.048	0.217	0.019	0.134
Left PT	0.225	0.019	0.024	0.175	0.018	1
Right vPreC	0.221	0.024	0.04	0.171	0.017	1
Right PT	0.235	0.019	0.002	0.185	0.012	0.998

Univariate fMRI results. **Figure S2** shows examples of two comparisons, including the contrast between all auditory events (both items and the probe of each trial) against fixation, as well as a contrast between activations to the visual retro cue and the visual "ready" cue that was presented at the onset of each task trial (**Fig. 1** of the main text).

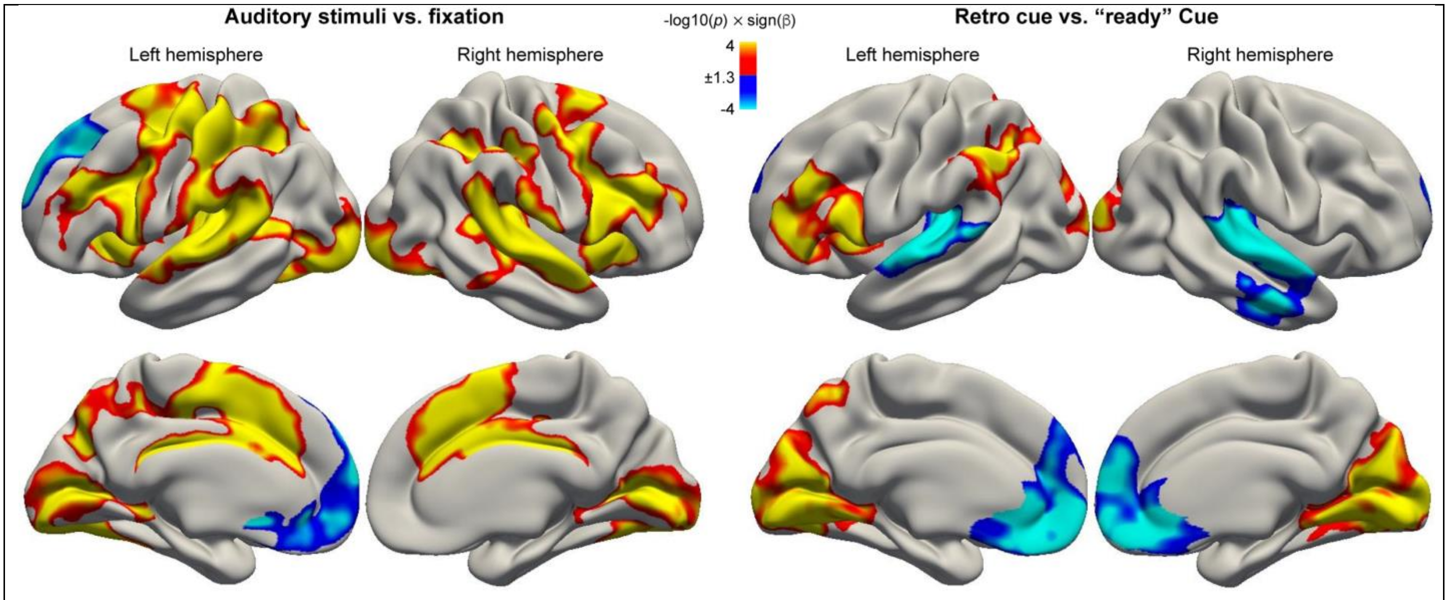

Supplementary Figure S2. Univariate GLM. (**Left**) GLM analysis contrasting all auditory stimuli, including items and probes, with fixation. (**Right**) Comparison between the retro cue and "ready cue". Significant increases of the BOLD signal were found in predominantly left hemispheric frontoparietal regions that were also involved in the functional connectivity patterns revealing WM content in our decoding analysis. The data show the statistical significance of the ininitial random-effects group analysis masked to the clusters with $p<0.05$ according to the permutation test.

Supplementary Methods

Univariate fMRI analysis. After cortical surface reconstructions and anatomical normalizations and distortion corrections, fMRI volume time series were motion corrected to common session-based template, realigned temporally to correct for the slice timing differences, coregistered with structural MRIs, intensity normalized, and resampled to the "fsaverage" standard brain surface representation. For the univariate analysis, the data were also smoothed along the surface, with iteration steps corresponding to a 8 mm full-width half maximum kernel. The data then were entered into a general-linear model (GLM) with the task conditions as explanatory variables.

The design matrix also included physiological and motion regressors of no interest, as well as polynomial regressors corresponding to a high-pass filter with a cutoff frequency of 0.006 Hz to remove low-frequency drifts in the BOLD signal. Random-effects group statistics were corrected for multiple comparisons using a cluster-based permutation test, with an initial cluster-forming threshold of $p>0.05$ and 1,000 permutations.

Supporting References

Benjamini, Y., Hochberg, Y., 1995. Controlling the False Discovery Rate: A Practical and Powerful Approach to Multiple Testing. Journal of the Royal Statistical Society 57, 289-300.
