# Supplementary material for: Spectrotemporal content of human auditory working memory represented in functional connectivity patterns
Source: Commun Biol. 2023 Mar 20;6:294. doi: 10.1038/s42003-023-04675-8 (PMC10027691; doi:10.1038/s42003-023-04675-8)
Supplement: Supplementary file 2 — Reporting Summary [file 42003_2023_4675_MOESM2_ESM.pdf]

nature portfolio

Corresponding author(s): Jyrki Ahveninen

Last updated by author(s): Sep 18, 2022

Reporting Summary

Nature Portfolio wishes to improve the reproducibility of the work that we publish. This form provides structure for consistency and transparency in reporting. For further information on Nature Portfolio policies, see our Editorial Policies and the Editorial Policy Checklist.

Statistics

For all statistical analyses, confirm that the following items are present in the figure legend, table legend, main text, or Methods section.

n/a	Confirmed	
☐	☒	The exact sample size (*n*) for each experimental group/condition, given as a discrete number and unit of measurement
☐	☒	A statement on whether measurements were taken from distinct samples or whether the same sample was measured repeatedly
☐	☒	The statistical test(s) used AND whether they are one- or two-sided<br>*Only common tests should be described solely by name; describe more complex techniques in the Methods section.*
☐	☒	A description of all covariates tested
☐	☒	A description of any assumptions or corrections, such as tests of normality and adjustment for multiple comparisons
☐	☒	A full description of the statistical parameters including central tendency (e.g. means) or other basic estimates (e.g. regression coefficient) AND variation (e.g. standard deviation) or associated estimates of uncertainty (e.g. confidence intervals)
☐	☒	For null hypothesis testing, the test statistic (e.g. *F*, *t*, *r*) with confidence intervals, effect sizes, degrees of freedom and *P* value noted<br>*Give P values as exact values whenever suitable.*
☒	☐	For Bayesian analysis, information on the choice of priors and Markov chain Monte Carlo settings
☒	☐	For hierarchical and complex designs, identification of the appropriate level for tests and full reporting of outcomes
☒	☐	Estimates of effect sizes (e.g. Cohen's *d*, Pearson's *r*), indicating how they were calculated

Our web collection on statistics for biologists contains articles on many of the points above.

Software and code

Policy information about availability of computer code

Data collection	Siemens MRI scanner software.
Data analysis	FreeSurfer, https://surfer.nmr.mgh.harvard.edu/fswiki<br>LIBSVM, https://www.csie.ntu.edu.tw/~cjlin/libsvm/<br>CoSMoMVPA toolbox, https://www.cosmomvpa.org/<br><br>The data and code for reproducing the connectivity-based main findings of the paper are available on Harvard Dataverse (https://doi.org/10.7910/DVN/51WR3A). All other data of this study are available from the corresponding author upon reasonable request.

For manuscripts utilizing custom algorithms or software that are central to the research but not yet described in published literature, software must be made available to editors and reviewers. We strongly encourage code deposition in a community repository (e.g. GitHub). See the Nature Portfolio guidelines for submitting code & software for further information.

Data

Policy information about availability of data

All manuscripts must include a data availability statement. This statement should provide the following information, where applicable:

- Accession codes, unique identifiers, or web links for publicly available datasets
- A description of any restrictions on data availability
- For clinical datasets or third party data, please ensure that the statement adheres to our policy

The data and code for reproducing the connectivity-based main findings of the paper are available on Harvard Dataverse (https://doi.org/10.7910/DVN/51WR3A). All other data of this study are available from the corresponding author upon reasonable request.

Human research participants

Policy information about studies involving human research participants and Sex and Gender in Research.

Reporting on sex and gender	The study was based on data from 20 healthy right-handed subjects (12 females, ages 22–47 years) with self-reported normal hearing. The data of two subjects of an initial sample of 23 were excluded due to the difficulty of perform the task (proportion correct 0.41 and 0.54), and one subject's data were excluded due to a triggering problem between the scanner and stimulus presentation computer. The protocol of the imaging experiment was approved by the Partners Human Research Committee, the Institutional Review Board (IRB) of the MGH. All subjects gave a written informed consent before participating in the study.
Population characteristics	The study was based on data from 20 healthy right-handed subjects (12 females, ages 22–47 years) with self-reported normal hearing.
Recruitment	Subjects were recruited through flyers and advertisements.
Ethics oversight	Mass General Brigham IRB

Note that full information on the approval of the study protocol must also be provided in the manuscript.

Field-specific reporting

Please select the one below that is the best fit for your research. If you are not sure, read the appropriate sections before making your selection.

☒ Life sciences ☐ Behavioural & social sciences ☐ Ecological, evolutionary & environmental sciences

For a reference copy of the document with all sections, see nature.com/documents/nr-reporting-summary-flat.pdf

Life sciences study design

All studies must disclose on these points even when the disclosure is negative.

Sample size	Selected based on previous fMRI studies in the field.
Data exclusions	The data of two subjects of an initial sample of 23 were excluded due to the difficulty of perform the task (proportion correct 0.41 and 0.54), and one subject's data were excluded due to a triggering problem between the scanner and stimulus presentation computer.
Replication	Within-participant leave one out crossvalidation.
Randomization	N/A, a within subjects design.
Blinding	N/A

Reporting for specific materials, systems and methods

We require information from authors about some types of materials, experimental systems and methods used in many studies. Here, indicate whether each material, system or method listed is relevant to your study. If you are not sure if a list item applies to your research, read the appropriate section before selecting a response.

Materials & experimental systems

n/a	Involved in the study
☒	☐ Antibodies
☒	☐ Eukaryotic cell lines
☒	☐ Palaeontology and archaeology
☒	☐ Animals and other organisms
☒	☐ Clinical data
☒	☐ Dual use research of concern

Methods

n/a	Involved in the study
☒	☐ ChIP-seq
☒	☐ Flow cytometry
☐	☒ MRI-based neuroimaging

Magnetic resonance imaging

Experimental design

Design type: Event-related task-based fMRI design.

Design specifications: . fMRI data were obtained with a gradient-echo (GE) EPI sequence, TR/TE = 1,470/30 ms, flip angle=82°, iPAT 2, SMS 3, 2×2×2 mm3 voxels; 69 axial slices. The acquisitions were obtained in 4 runs, each with 501 time points. In each run, there were 24 trials (4 trials per to-be-memorized item class).

Behavioral performance measures: Subjects were asked to indicate by pressing one of two buttons whether the probe sound matched the to-be-remembered item. Behavioral performance was determined as the proportion of correct responses.

Acquisition

Imaging type(s): High-resolution T1-weighted anatomical images were obtained using a multi-echo MPRAGE pulse sequence (TR=2530 ms; 4 echoes with TEs= 1.69, 3.55, 5.41, 7.27 ms; 176 sagittal slices with 1×1×1 mm3 voxels, 256×256 mm2 matrix; flip angle = 7°) (van der Kouwe et al., 2008) in a 3T Siemens Prisma whole-body MRI scanner (Siemens Medical Systems, Erlangen, Germany) using a 64-channel head and neck coil. fMRI data were obtained with a gradient-echo (GE) EPI sequence, TR/TE = 1,470/30 ms, flip angle=82°, iPAT 2, SMS 3, 2×2×2 mm3 voxels; 69 axial slices.

Field strength: 3T

Sequence & imaging parameters: High-resolution T1-weighted anatomical images were obtained using a multi-echo MPRAGE pulse sequence (TR=2530 ms; 4 echoes with TEs= 1.69, 3.55, 5.41, 7.27 ms; 176 sagittal slices with 1×1×1 mm3 voxels, 256×256 mm2 matrix; flip angle = 7°) (van der Kouwe et al., 2008) in a 3T Siemens Prisma whole-body MRI scanner (Siemens Medical Systems, Erlangen, Germany) using a 64-channel head and neck coil. fMRI data were obtained with a gradient-echo (GE) EPI sequence, TR/TE = 1,470/30 ms, flip angle=82°, iPAT 2, SMS 3, 2×2×2 mm3 voxels; 69 axial slices.

Area of acquisition: Whole head (see above)

Diffusion MRI: ☐ Used ☒ Not used

Preprocessing

Preprocessing software: Freesurfer FSFAST

Normalization: Using surface-based Freesurfer FSFAST tools. In the connectivity-based MVPA, ROIs determined in the standard space were resampled into each individual's functional space. In the activation-based MVPA, statistics were based on ROIs determined in the Freesurfer anatomical segmentation process.

Normalization template: Freesurfer surface-based "fsaverage" subject (icos 7 and 3).

Noise and artifact removal: fMRI functional connectivity patterns between the ROIs were based on the residuals of the task-related GLM calculated in each subjects native functional space, from which the nuisance effects (motion) and inter-regional co-activations related to task performance had been regressed out.

Volume censoring: N/A

Statistical modeling & inference

Model type and settings: All MVPA analyses were conducted using support vector machine (SVM) implemented in libsvm (Chang and Lin, 2011) and provided in the COSMOMVPA package (http://www.cosmomvpa.org/) (Oosterhof et al., 2016) in MATLAB. A SVM classifier with linear kernel and cost equal to one (C = 1) was trained using 18×T dataset and tested on 6×T dataset, employing four-fold cross validation. To control for multiple comparisons, statistical significances of decoding accuracies were tested at the group level using a nonparametric randomization approach. First, we created 500 random permutations where the true labels of the classifier were shuffled within each exchangeability block, i.e., the fMRI runs. To determine the classification accuracies that emerge by chance with 6-classes, a distribution of decoding accuracies using training data with randomized item-content labels was generated across all subjects and connectivity patterns (connectivity-based MVPA) or ROIs

(activation-based MVPA). For the final null distribution, we selected the maximum group mean across all possible connectivity patterns from each permutation. To assign a p-value for each connection, the original group mean accuracy value, found from classifiers with true labels, was compared with this null distribution.

Effect(s) tested

Define precise effect in terms of the task or stimulus conditions instead of psychological concepts and indicate whether ANOVA or factorial designs were used.

Specify type of analysis: ☐ Whole brain ☐ ROI-based ☒ Both

Anatomical location(s)

To test our connectivity-based hypothesis, we defined a set of broader ROIs reported previously to play a role in auditory or verbal WM (Mamashli et al., 2021; Uluc et al., 2018). The idea was that content-specific coding of WM information would be revealed based on the pattern of functional connectivity across the different subareas of these larger ROIs. Each of these broader ROIs was thus divided to multiple smaller subROIs whose average surface area across all subjects and areas was 157 mm2. It was our assumption that pooling together the signals to slightly larger subROIs would not only increase the computational efficiency and reduce the number of features in the decoding analysis, but also increase the SNR of the features.
Each subROI of the larger ROIs referred to the icosahedral patches corresponding to the vertices of the fsaverage3 standard brain (642 vertices / hemisphere), resampled to each individual subject's higher-resolution cortical representation. The ROIs included superior temporal cortex (STC; superior temporal gyrus and HG combined; nsubRois=34 left/29 right), middle frontal gyrus (MTG, rostral, caudal parts combined; nsubRois=43 left/50 right), inferior frontal gyrus (IFG; nsubRois=23 left/14 right), precentral cortex (PreC), supramarginal gyrus (SMG; nsubRois=34 left/32 right), angular gyrus (AG; nsubRois=33 left/37 right), and superior parietal lobule (SPL; nsubRois=76 left/80 right) (Fig. connROIs). In addition to the frontoparietal and temporal ROIs, we also included the occipital cortex (OC; nsubRois=62 left/51 right) to serve as a control area, with the assumption that this area would not play a major role in connectivity-based auditory WM maintenance (Mamashli et al., 2021).
The conventional MVPA analyses were conducted in the native functional space with no spatial smoothing: To focus the analyses to the cortical gray matter, and to minimize cross talk across sulci and gyri, a set of a priori anatomical regions-of-interest (ROI) were defined based on modified Freesurfer surface-based anatomical segmentations (Fig. 6). A total of 43 surface-based ROI labels per hemisphere were projected to each subject's unsmoothed native functional space. To define these areas, the Desikan anatomical parcellation (Desikan et al., 2006) was modified such that the combination of labels encompassing the superior temporal cortex (areas STG and HG) were divided to nine smaller parcels based on the more detailed Freesurfer Destrieux atlas (Destrieux et al., 2010), with the STG further divided to its anterior and posterior portions using mris_divide_parcellation. The STC areas of interest included Heschl's gyrus (HG), Heschl's sulcus (HS), planum temporal (PT), posterior STG (pSTG), anterior STG (aSTG), as well as the parts of Destrieux's lateral fissure (LF), inferior circular sulcus (iCS), and STS (STS/STG) that overlap with Desikan's STC. The more detailed parcellation of STC was utilized to pinpoint areas with sharpest auditory-parametric WM representations. In addition, we used mris_divide_parcellation to define the dorsal and ventral subareas of precentral (vPreC, dPreC) and postcenral areas (vPostC, dPostC) to search parametric WM representations specifically in the ventral sensorimotor areas that are presumed to involved in the "phonological loop" of auditory WM (Buchsbaum and D'Esposito, 2008).

Statistic type for inference
(See Eklund et al. 2016)

Maximum-statistic permutation testing (see above)

Correction

Maximum-statistic based robust non-parametric permutation testing.

Models & analysis

n/a	Involved in the study
☐	☒ Functional and/or effective connectivity
☒	☐ Graph analysis
☐	☒ Multivariate modeling or predictive analysis

Functional and/or effective connectivity

Pearson correlation matrices were used as the features for the SVM classifier.

Multivariate modeling and predictive analysis

All MVPA analyses were conducted using support vector machine (SVM) implemented in libsvm (Chang and Lin, 2011) and provided in the COSMOMVPA package (http://www.cosmomvpa.org/) (Oosterhof et al., 2016) in MATLAB. A SVM classifier with linear kernel and cost equal to one (C = 1) was trained using 18×T dataset and tested on 6×T dataset, employing four-fold cross validation, where T refers to the number of connectivity patterns (connectivity-based MVPA) or voxels in each ROI (activation-based MVPA), employing four-fold cross validation.
